# Apoptosis, autophagy, ferroptosis, and pyroptosis in cisplatin-induced ototoxicity and protective agents

**DOI:** 10.3389/fphar.2024.1430469

**Published:** 2024-09-24

**Authors:** Dingyuan Dai, Chao Chen, Chen Lu, Yu Guo, Qi Li, Chen Sun

**Affiliations:** ^1^ Department of Otolaryngology Head and Neck Surgery, Children’s Hospital of Nanjing Medical University, Nanjing, Jiangsu, China; ^2^ Nanjing Medical University, Nanjing, Jiangsu, China; ^3^ Medical School of Nanjing University, Nanjing, Jiangsu, China

**Keywords:** cisplatin, ototoxicity, apoptosis, autophagy, ferroptosis, pyroptosis, protective agents

## Abstract

Cisplatin is widely used to treat various solid tumors. However, its toxicity to normal tissues limits its clinical application, particularly due to its ototoxic effects, which can result in hearing loss in patients undergoing chemotherapy. While significant progress has been made in preclinical studies to elucidate the cellular and molecular mechanisms underlying cisplatin-induced ototoxicity (CIO), the precise mechanisms remain unclear. Moreover, the optimal protective agent for preventing or mitigating cisplatin-induced ototoxicity has yet to be identified. This review summarizes the current understanding of the roles of apoptosis, autophagy, ferroptosis, pyroptosis, and protective agents in cisplatin-induced ototoxicity. A deeper understanding of these cell death mechanisms in the inner ear, along with the protective agents, could facilitate the translation of these agents into clinical therapeutics, help identify new therapeutic targets, and provide novel strategies for cisplatin-based cancer treatment.

## 1 Introduction

Cisplatin is one of the most effective and widely used anticancer drugs. However, its use is limited by severe side effects, including ototoxicity, nephrotoxicity, neurotoxicity, and vascular toxicity (Ghosh, 2019). Among patients treated with cisplatin, 40%–80% of adults and at least 40% of children experience permanent hearing loss. Cisplatin-induced ototoxicity (CIO) typically presents as bilateral, progressive, irreversible, and dose-dependent sensorineural hearing loss, often accompanied by tinnitus and vertigo. CIO primarily affects the inner ear, targeting three main structures: the outer hair cells (OHCs) of the organ of Corti, spiral ganglion neurons (SGNs), and the stria vascularis (Marnitz et al., 2018; Waissbluth et al., 2017; Knight et al., 2017; Breglio et al., 2017; Frisina et al., 2016; van Ruijven et al., 2005). Despite extensive research, the molecular pathogenesis of CIO remains unclear, and effective strategies for its prevention and treatment have not yet been established. Given that mature mammalian inner ear cells have minimal regenerative capacity, understanding the mechanisms underlying CIO and developing effective preventive and therapeutic approaches are crucial.

Programmed cell death (PCD) is a genetically regulated, autonomous, and orderly process essential for maintaining cellular homeostasis. PCD involves the activation, expression, and regulation of specific genes and plays a significant role in various pathophysiological conditions, making it a key focus of recent research. The primary forms of PCD include apoptosis, ferroptosis, and pyroptosis. Although autophagy is often discussed in the context of cell death, it is better described as a cellular degradation and recycling process that promotes cell survival by eliminating damaged organelles, misfolded proteins, and other cellular debris. Unlike apoptosis and pyroptosis, which are more direct and structured forms of cell death, autophagy primarily functions to maintain cellular stability (Liu et al., 2023; Galluzzi et al., 2018). Oxidative stress is the primary mechanism of CIO and can modulate multiple PCD pathways simultaneously. Increasing evidence suggests that PCD plays a crucial role in CIO. In this review, we explore recent findings on the role of PCD pathways in CIO, particularly in the context of apoptosis, autophagy, ferroptosis, and pyroptosis, and discuss how these insights have advanced our understanding of the molecular mechanisms underlying CIO. Additionally, we summarize the protective agents identified for preventing and treating CIO to assist researchers in developing optimal strategies against this condition.

## 2 Apoptosis in CIO

### 2.1 Overview

Apoptosis is the most extensively studied cell death pathway associated with cisplatin-induced hearing loss and is considered the primary mode of cisplatin-induced damage to inner ear cells. Inhibition of apoptosis partially attenuates CIO.

Apoptosis is a form of programmed cell death that does not trigger an inflammatory response. A key feature of apoptosis is the activation of cysteinyl aspartate-specific proteinases (caspases). The apoptotic signal is transmitted through various signaling pathways, ultimately leading to caspase activation, which is responsible for cell death. Three main apoptotic signaling pathways have been identified: the intrinsic (mitochondrial) apoptosis pathway, the extrinsic (death receptor) apoptosis pathway, and the endoplasmic reticulum (ER) stress pathway. Although these pathways are distinct, they converge during the formation of apoptotic bodies, which are eventually engulfed by phagocytes (Xu et al., 2019; Green, 2019). All three apoptotic signaling pathways have been implicated in CIO, with the mitochondrial apoptosis pathway being the predominant pathway in cisplatin-induced apoptosis in the inner ear (Li et al., 2016).

### 2.2 Mitochondrial apoptosis pathway in CIO

Research has confirmed that cisplatin initiates mitochondrial apoptosis through three main mechanisms: 1) cisplatin induces the overproduction and accumulation of reactive oxygen species (ROS), which deplete glutathione and antioxidant enzymes; 2) cisplatin binds to and alkylates DNA, halting DNA replication and causing DNA damage; and 3) cisplatin decreases ATPase activity (Wang W. et al., 2022a). ROS accumulation is thought to be the primary mechanism by which cisplatin induces apoptosis in inner ear cells (Wang N. et al., 2022b; Li Y. et al., 2022a; Bu et al., 2022; Zheng et al., 2020).

**FIGURE 1:**
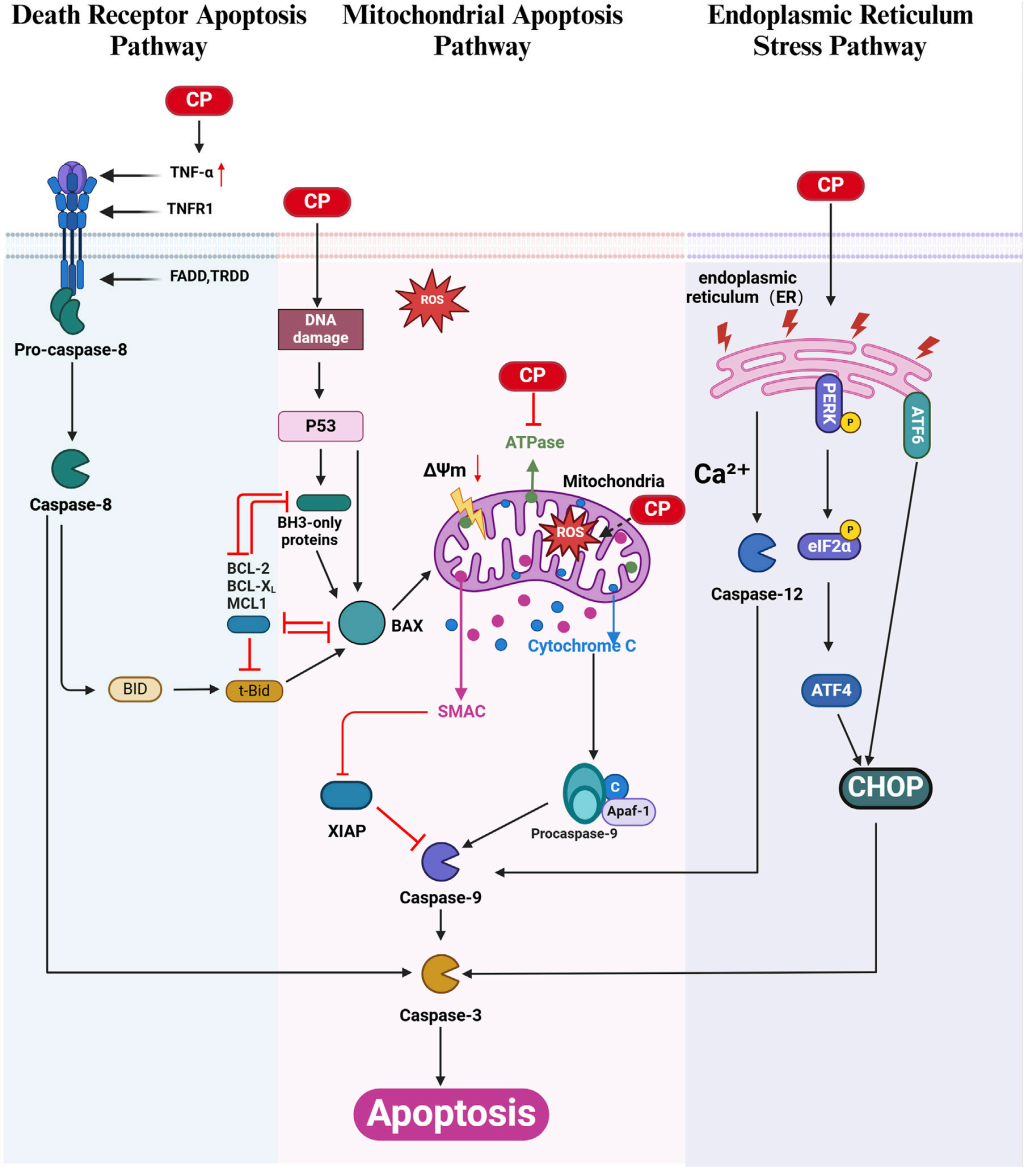
Apoptosis in CIO: Cisplatin (CP) triggers apoptosis through the extrinsic death receptor pathway (green), the mitochondrial intrinsic pathway (pink), and the endoplasmic reticulum (ER) stress pathway (purple). Cisplatin-induced DNA damage in cochlear cells can activate the mitochondrial apoptotic pathway *via* the p53 signaling cascade. Within the death receptor pathway, activated caspase-8 cleaves Bid to form tBid, which increases mitochondrial membrane permeability, linking the extrinsic and intrinsic apoptotic pathways. Cisplatin also induces ER stress by activating the caspase-12, PERK/eIF2α/CHOP, and ATF6/CHOP signaling pathways. The BCL-2 family proteins are categorized into anti-apoptotic proteins (including BCL-2, BCL-XL, and MCL-1) and pro-apoptotic proteins (including BH3-only, BAX, BID, and tBid). XIAP, an inhibitor of apoptosis, directly regulates cisplatin-induced cell death *via* caspase-9 modulation. (Created with BioRender.com).

Following cisplatin-induced ROS overproduction, DNA damage, and reduced ATPase activity in the inner ear, the expression of the proapoptotic protein Bax increases, while the expression of the antiapoptotic protein Bcl-2 decreases, leading to an increased Bax/Bcl-2 ratio. Bax is then translocated from the cytoplasm to the mitochondria, where it triggers a decrease in mitochondrial membrane potential (MMP, ΔΨm), increases mitochondrial membrane permeability, promotes Ca^2+^ influx, and releases cytochrome C (Cytc) into the cytoplasm. Cytc forms a multimeric complex with apoptotic protease Apaf-1, thereby activating the Caspase-9 precursor. Active Caspase-9 directly cleaves and activates the downstream effector Caspase-3, which in turn cleaves substrates, such as functional proteins, in inner ear cells, leading to apoptosis (Zhao et al., 2022; Xu et al., 2022; Lu X. et al., 2022a; He et al., 2022). Cisplatin-induced DNA damage also activates mitochondrial apoptosis via the p53 pathway (Wu et al., 2021). X-linked inhibitor of apoptosis protein (XIAP), a major member of the inhibitor of apoptosis protein (IAP) family, directly inhibits caspase-9-mediated apoptosis. Several studies have demonstrated that XIAP overexpression can partially protect against CIO (Li Y. et al., 2022a; Jie et al., 2015; Chan et al., 2007) (Figure 1).

### 2.3 Death receptor apoptosis pathway in CIO

Death receptors (DRs) belong to the tumor necrosis factor receptor (TNFR) superfamily. When a death receptor binds to its specific ligand, it receives an extracellular death signal that activates intracellular apoptotic mechanisms. Currently, three central apoptotic death receptor signaling pathways are recognized: Fas cell surface death receptor/Fas ligand (FAS/FASL), tumor necrosis factor-related apoptosis-inducing ligand receptor/tumor necrosis factor-related apoptosis-inducing ligand (TRAILR/TRAIL), and tumor necrosis factor receptor/tumor necrosis factor (TNFR/TNF) (Xu et al., 2019) (Figure 1).

Cisplatin upregulates the expression of tumor necrosis factor (TNF-α) (Kaur et al., 2011; Abi-Hachem et al., 2010; Jeong et al., 2007), which initiates the extrinsic apoptosis pathway by binding to members of the TNF receptor family. This interaction leads to the recruitment of proteins with death domain structures, such as Fas-associated death domain protein (FADD). The death effector domain of FADD interacts with procaspase-8, leading to its cleavage and activation. Activated caspase-8, in turn, activates downstream caspase-3, causing apoptosis in inner ear cells (Jeong et al., 2007; Lee HS. et al., 2020a; Ding et al., 2020). Additionally, at low concentrations, activated caspase-8 cleaves Bid, a proapoptotic member of the Bcl-2 family, resulting in the formation of truncated Bid (tBid), which increases mitochondrial membrane permeability and activates the mitochondrial apoptosis pathway (Abi-Hachem et al., 2010) (Figure 1).

### 2.4 Endoplasmic reticulum-stress pathway in CIO

Recent studies have highlighted the pivotal role of endoplasmic reticulum (ER) stress and the unfolded protein response (UPR) in cochlear cell apoptosis, a key event in hearing loss. Cisplatin disrupts proper protein folding or induces mistranslation, resulting in the accumulation of unfolded or misfolded proteins in the cochlear ER.

The ER is a crucial cellular organelle responsible for protein folding, calcium storage, and lipid synthesis. Under stress conditions induced by cisplatin, the ER’s capacity to properly fold proteins is overwhelmed, leading to the accumulation of unfolded or misfolded proteins. This accumulation triggers the UPR, a cellular stress response aimed at restoring ER homeostasis. However, persistent or excessive UPR activation can lead to apoptosis, particularly through specific signaling pathways within the UPR, such as the PERK/ATF4/CHOP and ATF6/CHOP pathways (Liu YC. et al., 2024a; Qu et al., 2023; Li J. et al., 2023a). Additionally, cisplatin upregulates the expression of active caspase-12 and caspase-9 in cochlear cells, further contributing to apoptosis via the ER stress pathway (Zong et al., 2017) (Figure 1).

The protein kinase RNA-like endoplasmic reticulum kinase (PERK) pathway is a key component of the UPR. Upon activation, PERK phosphorylates eukaryotic initiation factor 2α (eIF2α), leading to a global reduction in protein synthesis while selectively increasing the translation of activating transcription factor 4 (ATF4). ATF4 induces the expression of C/EBP homologous protein (CHOP), a transcription factor that promotes apoptosis. The PERK/ATF4/CHOP axis is critically involved in cisplatin-induced ototoxicity, with studies demonstrating a time-dependent increase in CHOP expression correlating with cochlear hair cell loss and elevated apoptosis *in vitro* and *in vivo* (Qu et al., 2023; Li J. et al., 2023a). Modulating the UPR to alleviate ER stress has emerged as a promising therapeutic approach. For instance, ISRIB, a small molecule that activates eIF2B and downregulates the PERK/CHOP pathway, protects against cisplatin-induced hearing loss without compromising the anticancer efficacy of the drug (Li J. et al., 2023a) (Figure 1).

Another critical factor in ER stress-mediated cisplatin ototoxicity is the activation of transcription factor 6 (ATF6). Unlike the PERK pathway, ATF6 activation typically restores ER function by upregulating chaperone proteins and components of the ER-associated degradation pathway. However, in the context of cisplatin exposure, ATF6 has been implicated in promoting apoptosis via CHOP regulation. Pharmacological activation of ATF6 in experimental models has been shown to mitigate hair cell apoptosis and preserve hearing, highlighting its dual role in both cell survival and death under stress (Liu YC. et al., 2024a) (Figure 1).

In addition to ATF6 and PERK, other UPR modulators have also been explored for their potential protective effects against cisplatin-induced ER stress. Salubrinal, an inhibitor of eIF2α dephosphorylation, has been reported to protect against cisplatin-induced cochlear hair cell death by maintaining eIF2α in its phosphorylated state, thereby reducing global protein synthesis and the burden of misfolded proteins within the ER (Lu W et al.). Similarly, tauroursodeoxycholic acid (TUDCA), a bile acid derivative, mitigated cisplatin-induced ototoxicity by inhibiting the accumulation of unfolded proteins and preventing ER stress-induced apoptosis (Wen et al., 2021; Lee CH. et al., 2020b).

In conclusion, the ER stress response plays a central role in the pathogenesis of cisplatin-induced ototoxicity. The PERK/ATF4/CHOP pathway, in particular, emerges as a critical mediator of cochlear cell apoptosis, and its modulation represents a promising therapeutic target. Further research into the precise mechanisms by which ER stress contributes to ototoxicity and the development of targeted therapies may pave the way for more effective prevention and treatment strategies for patients undergoing cisplatin chemotherapy.

## 3 Autophagy in CIO

### 3.1 Overview

Autophagy is a process through which eukaryotic cells utilize lysosomes to degrade cytoplasmic proteins and damaged organelles, regulated by autophagy-related genes (Atgs). This process prevents cellular damage, promotes cell survival during energy or nutrient deficiency, and responds to cytotoxic stimuli (Dikic and Elazar, 2018; Parzych and Klionsky, 2014). Autophagy serves as an essential survival pathway that supports cell growth and development, mitigates metabolic stress and oxidative damage, and plays a crucial role in maintaining intracellular homeostasis, as well as in the synthesis, degradation, and recycling of cellular components, contributing to cell death (Parzych and Klionsky, 2014; Murrow and Debnath, 2013). Numerous studies have indicated that autophagy is activated during cisplatin-induced ototoxicity. However, the precise role of autophagy in cisplatin-induced ototoxicity remains unclear; some studies suggest that enhanced autophagy may alleviate cisplatin-induced ototoxicity, while others propose that inhibiting autophagy could have a similar protective effect.

**FIGURE 2:**
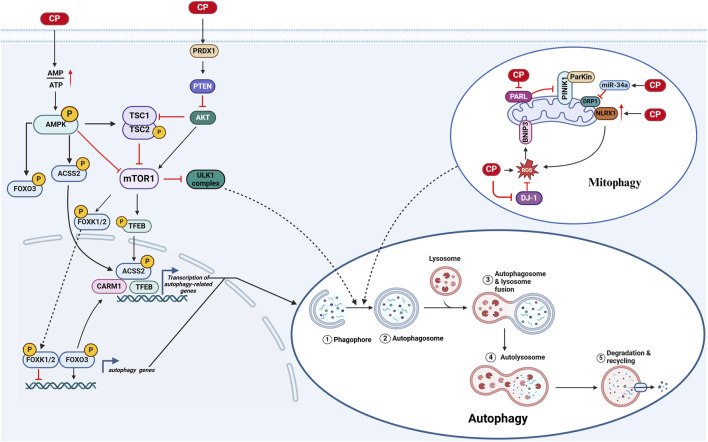
Autophagy in CIO: 1) Cisplatin decreases intracellular ATP levels, leading to the activation of autophagy through multiple pathways: **(A)** AMPK/mTOR1/ULK1 pathway: Activation of AMPK due to reduced ATP inhibits mTORC1, facilitating ULK1-mediated autophagy initiation; **(B)** AMPK/ACSS2/TFEB pathway: AMPK activation promotes the phosphorylation of ACSS2, enhancing TFEB nuclear translocation, which in turn upregulates autophagy and lysosomal biogenesis; **(C)** AMPK/FOXO3/CARM1 pathway: Activated AMPK phosphorylates FOXO3, promoting its activity and enhancing the expression of its target genes, including CARM1, further stimulating autophagy. 2) Cisplatin activates mitophagy through mechanisms involving oxidative stress: **(A)** BNIP3 activation: Cisplatin-induced oxidative stress increases BNIP3 levels, which promotes mitophagy; **(B)** PARL activity attenuation: Decreased activity of PARL leads to the stabilization of PINK1, recruiting Parkin to damaged mitochondria for degradation; **(C)** Increased NLRX1 activity: Cisplatin enhances NLRX1 expression, which facilitates the accumulation of LC3-II marked autophagosomes, promoting mitophagy in HEI-OC1 cells. (Created with BioRender.com).

Cisplatin activates autophagy in inner ear cells via several pathways: 1) A study has shown that cisplatin activates the PRDX1/PTEN/AKT signaling pathway, which stimulates autophagy in spiral ganglion neurons (SGNs) (Liu W. et al., 2021a). 2) Mitophagy in CIO: a) Cisplatin induces mitochondrial damage in inner ear cells, evidenced by a decrease in the activity of presenilin-associated rhomboid-like protease (PARL), leading to reduced degradation of phosphatase and tensin homolog-induced putative kinase 1 (PTEN-induced putative kinase 1, PINK1). This reduction stabilizes and recruits the E3 ubiquitin ligase Parkin to initiate autophagy (Cho et al., 2021; Yang et al., 2018; Onishi et al., 2021; Neumann et al., 2003). b) Cisplatin-induced oxidative stress, resulting from the inhibition of DJ-1 activity, increases the activity of Bcl-2/adenovirus E1B 19 kDa interacting protein 3 (BNIP3), a mitochondrial protein that promotes mitophagy (Cho et al., 2021; Wang Y. et al., 2023a). c) Nucleotide-binding domain and leucine-rich repeat containing family member X1 (NLRX1), a cytoplasmic pattern recognition receptor, has its expression increased by cisplatin, leading to the accumulation of LC3-II-labeled autophagosomes in HEI-OC1 cells. Furthermore, NLRX1 overexpression enhances mitochondria-derived reactive oxygen species (ROS) generation in response to cisplatin exposure, resulting in excessive autophagy activation (Yin et al., 2018). d) MicroRNAs (miRNAs) are a class of endogenous RNAs that are highly expressed in various cells of the animal cochlea and are closely associated with the development and pathological processes of the inner ear. Among these, miR-34a is involved in the regulation of cellular senescence, autophagy, and cell death. Dynamin-related protein 1 (DRP1) is a GTPase that plays a key role in initiating mitochondrial fission and mitophagy. Downregulation of DRP1 inhibits mitophagy, leading to the accumulation of damaged mitochondria. Studies have shown that cisplatin treatment increases the expression of miR-34a in C57BL/6 mice and HEI-OC1 cells while decreasing DRP1 levels, thereby inhibiting mitophagy (Wang H. et al., 2023b). 3) Cisplatin damages ATPases in inner ear cells, reducing ATP production and activating adenosine monophosphate-activated protein kinase (AMPK), which regulates the activity of the transcription factor forkhead box protein O3 (FOXO3) through direct phosphorylation. Additionally, AMPK reduces the activity of mammalian target of rapamycin complex 1 (mTORC1), leading to the dephosphorylation of mTORC1 and the targeting of FOXK1, FOXK2, and transcription factor EB (TFEB). Dephosphorylated FOXK1 and FOXK2 can no longer act as transcriptional repressors of FOXO3 target genes, allowing FOXO3 to bind to downstream autophagy genes and increase transcription. The dephosphorylation of TFEB causes its nuclear translocation and the activation of downstream target genes, including those involved in autophagy-related lysosomal biogenesis. Moreover, the increase in CARM1 protein levels induced by FOXO3-dependent gene activation further enhances the expression of TFEB-dependent genes, while the phosphorylation of acetyl-coenzyme A carboxylase 2 (ACC2) by AMPK stimulates the nuclear translocation of TFEB, increasing the expression of TFEB target genes (Li Z. et al., 2022b; Liang et al., 2021; Franco-Juarez et al., 2022). Furthermore, reduced mTORC1 activity weakens the inhibitory effect of the ULK1 complex, promoting autophagy. These findings underscore the intricate and multifaceted nature of autophagy regulation in cisplatin-induced ototoxicity, suggesting that while autophagy activation can serve as a protective response to cellular stress, its role is context-dependent and may lead to either cell survival or further damage, necessitating careful consideration in therapeutic strategies (Youn et al., 2015) (Figure 2).

### 3.2 Enhancing autophagy alleviates CIO

Research indicates that enhanced autophagy plays a crucial role in mitigating cisplatin-induced ototoxicity. For instance, glycogen synthase kinase 3 beta (GSK-3β), a serine/threonine kinase involved in various cellular processes such as metabolism and cell survival, negatively regulates autophagy; its inhibition promotes autophagy activation, thereby protecting against cisplatin-induced ototoxicity (Liu et al., 2019). Similarly, PINK1, a key mitochondrial quality control protein, accumulates in damaged mitochondria in response to cellular stress. This accumulation recruits the E3 ubiquitin ligase Parkin, initiating mitophagy, which removes damaged mitochondria and helps maintain mitochondrial health within cells. Studies have shown that PINK1 activation enhances autophagy in HEI-OC1 cells under cisplatin-induced stress and significantly reduces apoptosis (Yang et al., 2018). Furthermore, YTHDF1, a reader of m6A modifications, protects auditory hair cells from cisplatin-induced damage by facilitating the translation of ATG14 and activating autophagy (Huang et al., 2022). Pharmacological agents such as trehalose have also been shown to safeguard cochlear hair cells from cisplatin-induced harm by activating TFEB-mediated autophagy (Li Z. et al., 2022b). Moreover, metformin enhances autophagy through the upregulation of AMPK and FOXO3a, offering protective effects against cisplatin-induced ototoxicity (Liang et al., 2021). The autophagy inducer rapamycin increases levels of LC3-II and Beclin-1, alleviating cisplatin-induced ototoxicity *in vivo* and underscoring the protective role of autophagy in cisplatin-induced cellular damage (Fang and Xiao, 2014). This suggests that pharmacological or genetic strategies to enhance autophagy may provide new therapeutic avenues for treating CIO.

Several studies have shown that cisplatin exacerbates ototoxicity by inhibiting autophagy in auditory cells, further supporting the hypothesis that enhanced autophagy alleviates cisplatin-induced ototoxicity. For instance, Cho et al. demonstrated that inhibiting mitophagy intensifies the damage caused by cisplatin in HEI-OC1 cells, while the autophagy activator CCP protects these cells by accelerating mitophagy (Cho et al., 2021). Additionally, other studies have indicated that cisplatin increases the expression of miR-34a in C57BL/6 mice and HEI-OC1 cells, which suppresses DRP1 levels, further inhibiting mitophagy and leading to increased cellular damage (Wang H. et al., 2023b).

In summary, these studies collectively highlight the crucial role of autophagy in protection against cisplatin-induced ototoxicity and suggest potential therapeutic avenues for enhancing autophagy in clinical settings.

### 3.3 Inhibiting autophagy alleviates CIO

Inhibition of autophagy has shown significant potential in reducing cisplatin-induced ototoxicity. Several studies have demonstrated this, including the use of the autophagy inhibitor LY294002, which effectively reduces cisplatin-induced apoptosis. Similarly, meclofenamic acid (MA2), a non-steroidal anti-inflammatory drug (NSAID) known for inhibiting cyclooxygenase enzymes, decreases cisplatin-induced cellular damage by inhibiting excessive autophagy (Li et al., 2018). Moreover, increased expression of NLRX1 in cisplatin-treated HEI-OC1 cells enhanced autophagy activation, leading to elevated mitochondria-derived reactive oxygen species (ROS) and increased cell death (Yin et al., 2018). Collectively, these findings suggest that autophagy inhibition may be an effective strategy for mitigating cisplatin-induced ototoxicity.

### 3.4 Autophagy is a double-edged sword

Autophagy may act as a double-edged sword in cisplatin-induced ototoxicity. One previous study indicated that inhibiting autophagy by upregulating the class III PI3K pathway protects HEI-OC1 cells during the early stages of cisplatin treatment. However, in later stages, increased autophagy activation through the inhibition of the mTOR pathway leads to cell death. Additionally, the study demonstrated that pretreatment with hydroxychloroquine, a lysosomal chloroquine derivative, inhibited lysosomal acidification and blocked autophagy, thereby protecting auditory cells from cisplatin-induced cytotoxicity. This further supports the notion that autophagy promotes cell death during the later stages of cisplatin exposure. These findings suggest that autophagy may play distinct roles during different stages of cisplatin treatment (Youn et al., 2015).

Previous studies have consistently shown that the number of cell deaths increases with the duration of cisplatin-induced damage. We hypothesized that in the early stages of cisplatin-induced damage, upregulation of autophagy may promote cell survival by clearing oxidative stress-induced damaged mitochondria and aberrant proteins. Conversely, in later stages, excessive autophagy could lead to cell death due to the accumulation of reactive oxygen species (ROS), damaged mitochondria, and proteins. It is important to note that differences in experimental models and research conditions could affect the generalizability of these results. For instance, the aforementioned study revealed that inhibiting autophagy via the activation of the class III PI3K pathway during the early stages of cisplatin treatment protected auditory cells, whereas in the later stages, increased autophagy activation through mTOR inhibition led to cell death. This indicates that the role of autophagy depends on specific cellular contexts, signaling pathways, and experimental conditions. Therefore, further research is needed to elucidate the mechanisms underlying autophagy under various conditions to better understand its complex role in cisplatin-induced ototoxicity.

In conclusion, autophagy in cisplatin-induced ototoxicity may exert both protective and pro-apoptotic effects depending on the timing and severity of cellular damage. This dual mechanism offers a new perspective for clinical treatment, suggesting that the timing and degree of autophagy regulation during cisplatin therapy may be crucial for mitigating ototoxicity.

## 4 Ferroptosis in CIO

Recent studies have shown that ferroptosis plays a significant role in cisplatin-induced ototoxicity. Ferroptosis is an iron-dependent form of programmed cell death characterized by the accumulation of lipid peroxides beyond the cell’s antioxidant capacity (Tang D. et al., 2021a). Cisplatin can increase the iron load and lipid peroxides in inner ear cells, leading to ferroptosis in cochlear hair cells and subsequent hearing loss. The inhibition of ferroptosis signaling pathways has been shown to mitigate cisplatin ototoxicity.

**FIGURE 3:**
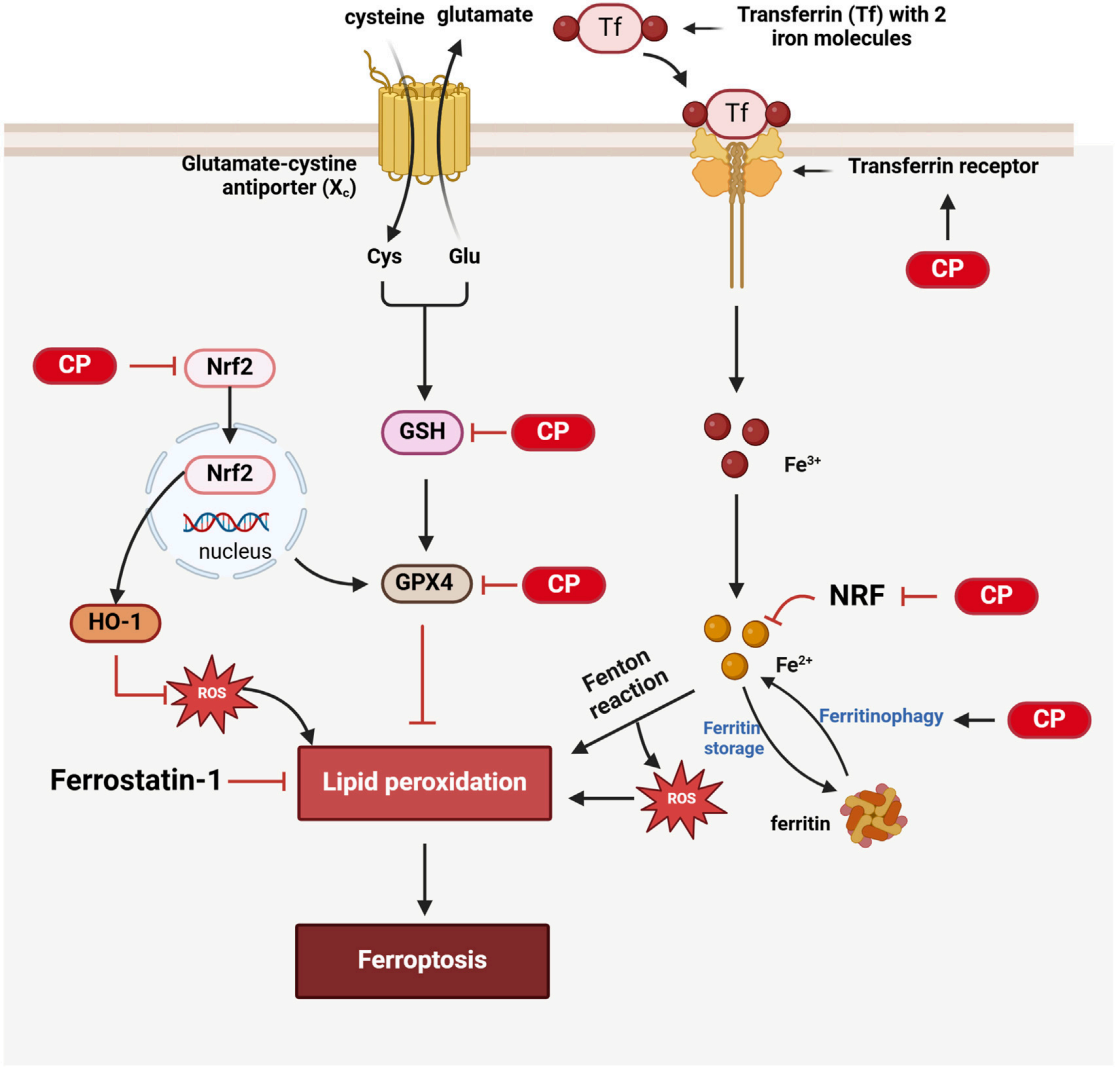
Ferroptosis in CIO: 1) Cisplatin inhibits GSH and GPX4, resulting in lipid peroxidation and subsequent ferroptosis. 2) Cisplatin activates ferritinophagy, leading to the release of Fe^2^⁺ from ferritin and the generation of reactive oxygen species (ROS) *via* the Fenton reaction, contributing to lipid peroxidation; additionally, Nrf2 inhibits this process, while cisplatin also suppresses Nrf2. 3) Ferrostain-1 can inhibit ferroptosis by reducing lipid peroxidation. 4) Cisplatin suppresses the Nrf2/HO-1 antioxidant pathway, resulting in oxidative stress that promotes lipid peroxidation and triggers ferroptosis. (Created with BioRender.com).

Recent studies have indicated that ferroptosis in cisplatin-induced ototoxicity primarily acts through the regulation of nuclear factor erythroid 2-related factor 2 (NRF2) and glutathione peroxidase 4 (GPX4) signaling pathways. NRF2 is a key transcription factor whose activation enhances cellular antioxidant capacity. Activation of NRF2 can upregulate antioxidant genes such as heme oxygenase-1 (HO-1) and inhibit ferroptosis. For example, the NRF2-specific activator 4-octyl itaconate significantly reduces cisplatin-induced ferroptosis by activating the NRF2/HO-1 signaling pathway (Zhang et al., 2024). Plant components, such as nobiletin, have also been shown to alleviate cisplatin ototoxicity through similar mechanisms (Song et al., 2024). Cisplatin induces ferroptosis in inner ear hair cells by inhibiting GPX4 activity, leading to lipid peroxidation. Antioxidants such as nobiletin and alpha-lipoic acid significantly reduce cisplatin-induced ototoxicity by activating GPX4 and reducing lipid peroxidation (Song et al., 2024; Cho et al., 2022). Furthermore, severe outer hair cell loss and progressive inner hair cell synapse damage were observed in GPX4 (−/−) mice, further demonstrating the critical role of GPX4 in maintaining inner ear hair cell function. Studies have also found that ferroptosis biomarkers, such as transferrin receptor 1 (TfR1), are upregulated in the outer hair cells of cisplatin-treated mice, and changes in multiple ferroptosis-regulating genes suggest that cisplatin-induced ototoxicity is closely related to ferroptosis (Liu Z. et al., 2024b). Additionally, the use of ferroptosis inhibitors, such as Ferrostatin-1, can significantly reduce cisplatin-induced ototoxicity by inhibiting lipid peroxidation and iron accumulation, thereby protecting cochlear cells from damage, further supporting the critical role of ferroptosis in cisplatin ototoxicity (Mei et al., 2020; Hu et al., 2020) (Figure 3).

Notably, ferroptosis and autophagy are closely related. Autophagy can clear damaged cellular components and influence ferroptosis by regulating iron and lipid metabolism. Ferroptosis depends on redox-active iron, which is primarily stored in cells as ferritin. Autophagy can promote ferritin degradation, releasing redox-active iron and ultimately inducing ferroptosis, a process known as ferritinophagy (Gao et al., 2016). Studies have found that autophagy-dependent ferroptosis plays an important role in cisplatin-induced ototoxicity, and autophagy inhibitors can significantly reduce cisplatin-induced inner ear damage (Jian et al., 2021; Zhou et al., 2020) (Figure 3).

Ferroptosis plays a key role in cisplatin-induced ototoxicity. By regulating the NRF2 and GPX4 signaling pathways and the interplay between autophagy and ferroptosis, further research into this mechanism provides potential targets for developing new strategies to protect cochlear cells, offering new perspectives for clinical applications.

## 5 Pyroptosis in CIO

Pyroptosis is a form of cell death mediated by the gasdermin (GSDM) family of proteins, such as GSDMD and GSDME. It is characterized by the activation of the NOD-like receptor protein 3 (NLRP3) inflammasome, formation of cell membrane pores, and release of interleukin (IL)-1β and IL-18 (Kuang et al., 2017; Shi et al., 2015). The GSDMD N-terminal structural domain (GSDMD-N) is generated by the cleavage of GSDMD proteins by pro-inflammatory caspases through classical and non-classical inflammasome signaling pathways. This cleavage leads to the rapid insertion of GSDMD-N into the plasma membrane, forming active pores that release cytokines, resulting in intense inflammation and cell death (de Vasconcelos et al., 2019; Xu et al., 2018). The NLRP3 inflammasome, a prominent multiprotein complex, consists of NLRP3, apoptosis-associated speck-like protein (ASC), and pro-caspase-1 (Sun et al., 2019). Cisplatin exerts ototoxic effects through pyroptosis, and inhibiting this process has been shown to alleviate cisplatin-induced hearing loss (Yu W. et al., 2022a; Yu R. et al., 2022b; Li et al., 2021).

**FIGURE 4:**
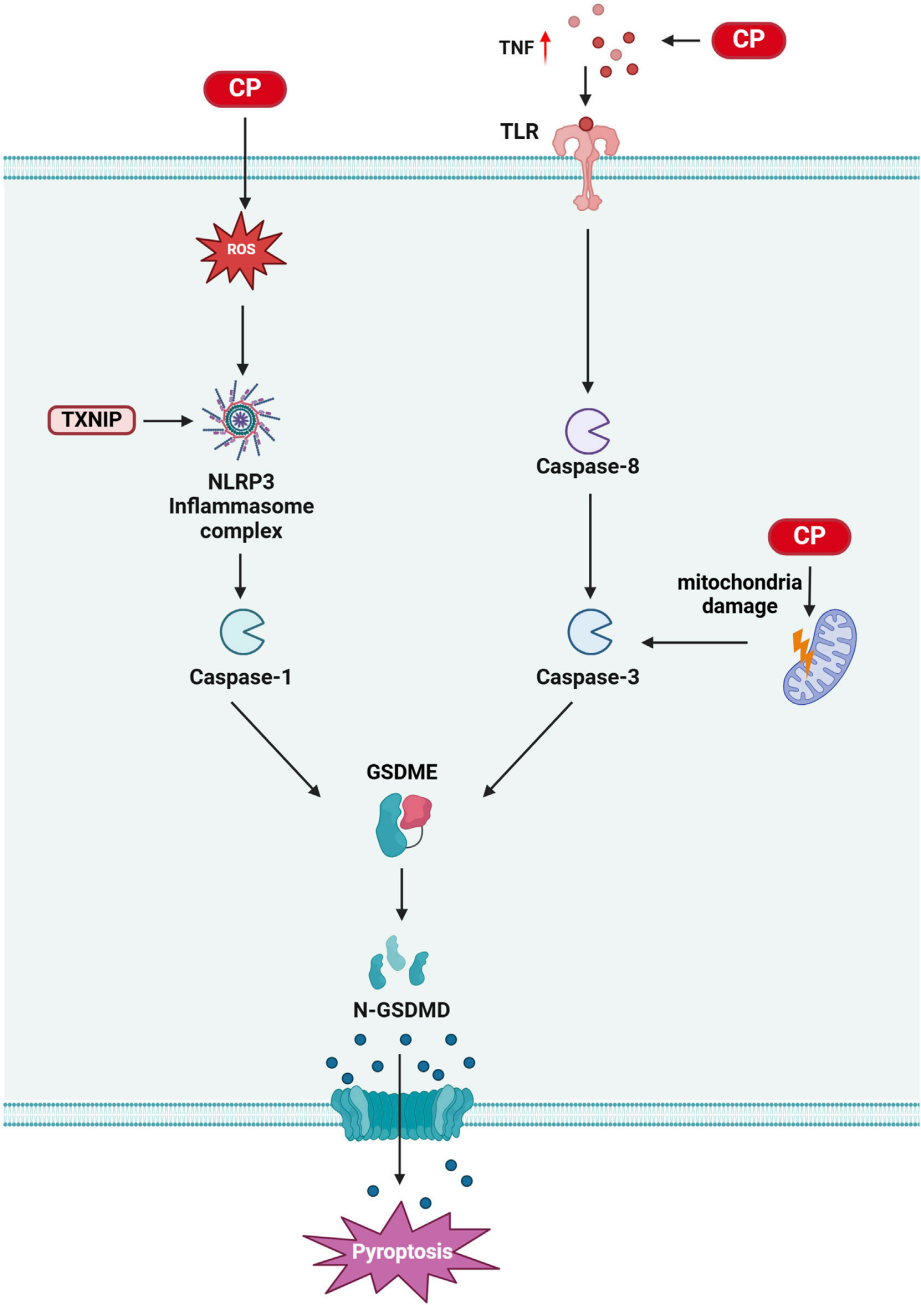
Pyroptosis in CIO: Cisplatin treatment leads to elevated intracellular reactive oxygen species (ROS), which activate the NLRP3/Caspase-3/GSDME pathway, resulting in pyroptosis. Additionally, cisplatin treatment induces an increase in tumor necrosis factor (TNF), causes mitochondrial damage, and activates caspase-3. This activation, in turn, triggers GSDME, which mediates cytosolic pyroptosis. (Created with BioRender.com).

Thioredoxin-interacting protein (TXNIP) is an α-inhibitory protein essential for redox homeostasis, primarily inducing apoptosis or pyroptosis under oxidative stress conditions (Pan et al., 2022). Studies indicate that inhibiting TXNIP expression or the dissociation of thioredoxin (Trx) from TXNIP can reduce NLRP3 activation and inhibit pyroptosis onset (Jia et al., 2020; An et al., 2020; Heo et al., 2019). A recent study demonstrated that NLRP3 inflammasome-mediated cell death is one mechanism by which cisplatin induces cochlear marginal cell (MC) injury. Cisplatin increases the expression of NLRP3 inflammasome components in MCs, and downregulating TXNIP inhibits cisplatin-induced NLRP3 inflammasome-mediated MC death. Furthermore, mutations in the Pou4f3 gene promote autophagy and apoptosis in cochlear hair cells from cisplatin-induced deaf mice, with recent findings showing that these mutations also activate the NLRP3/Caspase-3/GSDME pathway, leading to pyroptosis (Yu R. et al., 2022b). Notably, compounds such as naringin and N-acetylcysteine can inhibit cisplatin-induced hair cell pyroptosis, providing partial alleviation of cisplatin-induced hearing loss (Wang W. et al., 2022a; Li et al., 2021) (Figure 4).

## 6 Protective agents against CIO

Substantial evidence supports the protective effects of various compounds against cisplatin-induced loss of cochlear cells in experimental models. These models, including mice, hair cell lines, and zebrafish, are crucial for elucidating the mechanisms of ototoxicity and exploring the pathways involved in cisplatin-induced hearing loss. Each model has unique advantages: the mouse model allows for in-depth analysis of genetic and physiological factors, whereas zebrafish provide a transparent system for real-time observation of cellular responses (Li KH. et al., 2024a; Lyu et al., 2023). By combining insights from these models, researchers can gain a comprehensive understanding of the effects of cisplatin on cochlear cells. When investigating the mechanisms of cell death, the choice of administration route and dosage is critical. Different administration methods, such as intravenous injection or direct addition to culture media, can significantly influence the drug concentration within cells or tissues. This, in turn, affects cell survival and death, enabling researchers to define thresholds for drug-induced apoptosis or necrosis. For example, in mouse models, the dose and frequency of intraperitoneal cisplatin injections significantly affect cell death (Ingersoll et al., 2023; Perše, 2021), whereas zebrafish models highlight early cellular damage due to varying exposure times and concentrations (Li KH. et al., 2024a; Domarecka et al., 2020). Such insights not only clarify the underlying mechanisms of cisplatin-induced ototoxicity but also lay the groundwork for developing protective strategies (Tang Q. et al., 2021b; Yu et al., 2020). In summary, the selection of appropriate experimental models is essential for understanding cisplatin-induced cell death. These models not only aid in uncovering the mechanisms of ototoxicity but also provide valuable experimental evidence for identifying effective intervention measures.

In September 2022, sodium thiosulfate received its first approval in the USA for reducing the risk of ototoxicity associated with cisplatin in pediatric patients aged 1 month and older with localized, non-metastatic solid tumors (Dhillon, 2023). Other agents, such as amifostine, a thiol-reducing agent and potent free-radical scavenger with demonstrated otoprotective properties against cisplatin in experiments using hamsters and guinea pigs, and atorvastatin, a hydroxymethylglutaryl-CoA (HMG-CoA) reductase inhibitor, have been assessed in clinical trials (Gurney et al., 2014; Fernandez et al., 2021). However, some studies reported contradictory results or unsatisfactory protection. In this review, we summarize the compounds that have been shown to protect against CIO in current preclinical studies, within the context of cell death and the mechanisms regulating cell death, to provide a reference for researchers. As shown in the tables, most of the related studies have focused on attenuating cisplatin-induced apoptosis and developing compounds that protect against CIO. Few studies have evaluated autophagy and ferroptosis, and no protective compounds have been developed based on other forms of cell death (Tables 1–3).

**TABLE 1:** CIO protective agents associated with apoptosis.

Compound	Target	Mechanism	Model	Apoptosis	Effect	Reference	Year
FPS-ZM1 (a RAGE inhibitor)	IκBα↑,p-IκBα↓, p-NF-κB (in the nucleus) ↓,COX-2↓,IL-1β↓,TNF-α↓,Cleaved-caspase 3↓,ROS↓,Bax↓,Bcl-2↑,p-p38↓,p-JNK↓,p-ERK↓,p-c-jun↓, Serum MDA↓, Serum SOD ↑	HMGB1/RAGE	C57BL/6 mice	Decrease	Protection	Qiao et al. (2024)	2024
Hesperidin	Cleaved-caspase 3↓,cleaved-PARP↓,ROS↓,Nrf2↑,NQO1↑	Nrf2/NQO1	C57BL/6j mice,HEI-OC1 cell	Decrease	Protection	Lou et al. (2024)	2024
Schisandrin B	ROS↓,Cleaved-caspase 3↓	/	C57BL/6j mice,HEI-OC1 cell	Decrease	Protection	Li et al. (2024b)	2024
AdipoRon (AR)	Cleaved-caspase 3↓,Bcl-2↑,ROS↓, AdipoR 1↑,SIRT1 ↑,TFAM↑	AdipoR 1	HEI- OC1 cell, C57BL/6mice	Decrease	Protection	Nong et al. (2023)	2023
LLY-283	PRMT5↓,ROS↓,Bax↓,Bcl- 2↑,Bax/Bcl- 2 ↓,Cleaved -PARP↓,Cleaved -caspase-3 ↓,H4R3me2s↓,H3R8me2s↓,Trp53↓,Bad↓,PI3K↑,p-PI3K↑,p-AKT↑	PI3K/AKT	HEI- OC1 cell, C57BL/6mice	Decrease	Protection	Zhao et al. (2022), Zheng et al. (2023)	2023 2022
RG108	Bax↓, BCL2↑, Caspase-3↓,p-PI3K↑, p-AKT↑, Nrf2↑,HO-1↑, NQO1↑	Nrf2/HO-1/NQO1, PI3K/Akt	HEI-OC1 cell	Decrease	Protection	Zhang et al. (2023)	2023
Apelin-13	ROS↓,Cleaved-caspase 3↓,Bcl-2↑,TNF-a↓,IL-6↓,p-STAT1↑,p-STAT3↓	STAT1 STAT3	C57BL/6mice	Decrease	Protection	Yin et al. (2023)	2023
Cinchonine (CN) and cinchonidine (CD)	Cleaved-caspase 3↓,ROS↓,p-PI3K↑,p-AKT↑	PI3K/AKT	HEI- OC1 cell, C57BL/6mice	Decrease	Protection	Tang et al. (2023)	2023
20(S)-Ginsenoside Rh1	ROS↓,Cleaved- caspase 3↓,p-p38↓,p-JNK↓	MAPK	HEI- OC1 cell, C57BL/6 mice	Decrease	Protection	Qiao et al. (2023)	2023
ISRIB	eIF2a↑,ATF4↓,Chop↓,DR5 ↓	PERK/CHOP	Balb/cJ mice,C57BL/6J mice	Decrease	Protection	Li et al. (2023a)	2023
Puerarin	Bax↓,Cleaved -caspase-3↓,ROS↓,pAKT↑,TRPV1↓,IP3R1↓,p65↓	Akt,TRPV1/IP3R1/p65	HEI- OC1 cell, C57BL/6 mice	Decrease	Protection	Xu et al. (2022), Lin et al. (2023)	2022 2023
5,7-Dihydroxy-4-methylcoumarin (D4M)	ROS↓,Cleaved -caspase-3↓,p-JNK↓,p-FoxO1/FoxO1↑	JNK/FoxO1	HEI- OC1 cell, C57BL/6 mice	Decrease	Protection	Li et al. (2023b)	2023
Aucubin	ROS↓,Cleaved-caspase-3↓,Bcl- 2↑,p-STAT3↑,p-AKT↑,PI3K↑	PI3K/AKT/STAT3	HEI- OC1 cell C57BL/6J mice	Decrease	Protection	Jiang et al. (2023)	2023
Esomeprazole	OCT2↓,TUNEL-positive cells↓	OCT2	HEI- OC1 cell	Decrease	Protection	Jeon et al. (2023)	2023
Fucoidan	ROS↓,Bcl- 2↑,Bax↓,Cleaved-caspase-3 ↓,Nrf2↑,Cleaved-caspase-9↓,HO-1↑,Cleaved-PARP↓,NQO1↑,SOD1↑,SOD2↑,GPx↑	Nrf2/HO-1	UB/OC-2 cell	Decrease	Protection	Hsieh et al. (2023)	2023
N-acetylcysteine (NAC)	ROS↓,GSH↑,NO↓	/	Sprague-Dawley rat, zebrafish	Decrease	Protection	Wang et al. (2022a), Somdaş et al. (2020)	2022
Melatonin	Bax↓,Caspase-3↓,Bcl- 2↑,Caspase-9↓	/	HEI-OC1 cell, C57BL/6 mice	Decrease	Protection	Wang et al. (2022b), Fernandez et al. (2015)	2022 2015
Astaxanthine	ROS↓,Cleaved-caspase-3↓,Caspase-8↓,Fadd↓,Bax↓,Caspase-3↓,Caspase-9↓,Bcl-2↑	NRF2	FVB breeding mice, C57BL/6 mice, HEI-OC1 cell	Decrease	Protection	Nan et al. (2022)	2022
Euppatilin	ROS↓,Bax↓,Bax/Bcl-2↓,caspase-3↓,PARP↓,p- p38 ↓,JNK↓	p38/JNK	HEI-OC1 cell,The trans-genic zebrafish lineTg (Brn3C:EGFP), C57BL/6 mice	Decrease	Protection	Lu et al. (2022a)	2022
Salubrinal	Cleaved-caspase 3↓,CHOP↓,Cleaved- PARP↓,BIP↑,p-eIF2α↑	eIF2a	C57BL/6 mice, HEI-OC1 cell	Decrease	Protection	Lu et al. (2022b)	2022
CPI-455	KDM5A↓,ROS↓,p-p38↓,p-JNK↓,BAX↓,Cleaved- caspase-3↓,H3K4 Tri-methylation↑	KDM5A, MAPK and PI3K/AKT	P2 wild-type mice, C57BL/6 mice	Decrease	Protection	Liu et al. (2022)	2022
ATX-PPS-NP	ROS↓,IL-6↓,GSH↑,4-HNE↓,Cytochrome-C↓,Cleaved-caspase 3↓	/	HEI-OC1 cell, C57BL/6 mice	Decrease	Protection	Gu et al. (2022)	2022
Tauroursodeoxycholic acid (TUDCA)	Cleaved-caspase-12↓,CHOP↓,UGGT1↓,OS9↓,HO1↓,SOD2↓,Cleaved- caspase 3↓,ROS ↓, iNOS ↓,Cleaved-caspase 3↓	HO1,CHOP	Sprague-Dawley rat, HEI-OC1 cell	Decrease	Protection	Wen et al. (2021), Lee et al. (2020b), Shah et al. (2020)	2021 2020 2020
U0126	Cleaved -caspase-3↓,ROS↓,MMP↑,p-ERK1/2 ↓,γH2AX↓	ERK	wild-type C57BL/6 mice, HEI-OC1 cell	Decrease	Protection	Wang et al. (2021)	2021
Resveratrol	ROS↓,CAT activity↑,PTEN↓,miR-455-5p↑,p-AKT↑,p-PI3K↑	PTEN-PI3K-Akt	HEI-OC1 cell, C57BL/6J mice	Decrease	Protection	Liu et al. (2021b)	2021
Naringin (Nar)	ROS↓,p53↓,Bax↓,Caspase-3↓	/	zebrafish	Decrease	Protection	Li et al. (2021)	2021
	
salvianolic acid B(Sal B)	Cleaved-caspase 3 ↓,ROS↓,Bcl-2↑,Bax↓,p-PI3K↑,p-Akt↑	PI3K-Akt	HEI-OC1 cell, Zebrafish	Decrease	Protection	Zheng et al. (2020)	2020
Hydroxytyrosol (HT)	p-JNK↑,Cleaved-caspase 3↑,AIF↑	JNK,AIF	C57BL/6 mice, HEI-OC1 cell	Increase	Injury	Zhang et al. (2020)	2020
Apelin	ROS↓,Cleaved-caspase3↓,p-JNK↓,Cleaved caspase-9↓,Bax↓	JNK	C57BL/6 mice , HEI-OC1 cell	Decrease	Protection	Yin et al. (2020)	2020
CYM-5478	ROS↓,Bax↓,P-Stat3↑,S1P2↑,Bcl-xL↑,Caspase- 3↓,Caspase-7↓	S1P2	Zebrafish, S1P2 Knockout Mice; S1pr2^−/−^ mice, C6 rat glioma cell, MDA-MB-231 cell	Decrease	Protection	Wang et al. (2020), Herr et al. (2016)	2020 2016
2-Isopropyl-3H-naphtho (1,2-d)imidazole-4,5-dione (KL1333)	ROS↓,Caspase-3↓	/	Institute for Cancer Research (ICR) mice	Decrease	Protection	Lee et al. (2020a)	2020
EPZ020411 (a selective small molecule PRMT6 inhibitor)	Cleaved-caspase-3 ↓,ROS↓,Cytochrome-translocation↓	PRMT6	C57BL/6 mice, HEI-OC1 cell	Decrease	Protection	He et al. (2020)	2020
Tetramethylpyrazine (Tet)	FoXo3↓,Bcl-2 binding ↓,component 3 (BBC3)↓,iGF1↑,TCF7L1↑,FZD6↑	Wnt,IGF1, FoXo3	HEI-OC1 cell	Decrease	Protection	Guan et al. (2020)	2020
Panax notoginseng Saponins (PnS)	ROS↓,Nrf2↑,NQo1↑,Ho-1↑,Gclc↑,p-AKT↑	AKT/Nrf2/HO-1	HEI-OC1 cell	Decrease	Protection	Fei et al. (2020)	2020
paeoniflorin (PF)	ROS↓,PINK1↑,BAD↓,Bax↓,Cleaved caspase-3 ↓	PINK1	C57BL/6 mice	Decrease	Protection	Yu et al. (2019)	2019
C-phycocyanin (C-PC)	ROS↓,Bax↓,Bcl-2↑,ATP↑,Caspase-9↓,Caspase-3↓	/	HEI-OC1 cell	Decrease	Protection	Kim et al. (2019)	2019
Ferulic acid	ROS↓,Cleaved-caspase-3 ↓,Cleaved-PARP↓,Nrf2↑	Nrf2	HEI-OC1 cell, C57BL/6 mice	Decrease	Protection	Jo et al. (2019)	2019
Allicin	Cleaved caspase-3↓,PARP-1↓,AIF ↓,Bax↓,Cleaved-caspase-9↓,Cleaved-caspase-3↓,cytochrome c↓,Bcl-2↑,p53↓,MDA↓,SOD↑	AIF,p53	C57 mice	Decrease	Protection	Cai et al. (2019), Wu et al. (2017)	2019 2017
Forskolin (FSK)	cAMP↑,ROS↓,Bax↓,Caspase-3↓,Bcl-2↑	PKA/MAPK	HEI-OC1 cell, C57BL/6J mice	Decrease	Protection	Guo et al. (2018)	2018
2-methyl-1-propyl-1H-indol-3-yl)-1–45naphthalenylmethanone (JWH015)	ROS↓,Caspase-3↓,Caspase-8↓,Caspase-9↓,TNF-a↓	CB2R	Mice expressing the CB2R tagged with green fluorescent protein (GFP), Wistar rat, UB/OC-1 cell,HEI-OC1 cell	Decrease	Protection	Jeong et al. (2007), Ghosh et al. (2018)	2018 2007
Peanut sprout extract (PSE)	Cleaved-PARP↓,Cleaved-caspase 3↓,Bcl-2↑,Caspase-3 ↓,PARP↓,ROS↓,NQO1↑,HO-1↑,GPx2↑,Gclc↑,Catalase↑,Nrf2↑,P-Akt↑	Akt/Nrf2	HEI-OC1 cell	Decrease	Protection	Youn et al. (2017)	2017
EpigallocatechiN-3-gallate (EGCG)	p53↓,Cleaved-caspase-3↓,Bax↓,Bcl-xL↑,ROS↓,p-ERK1/2↓,p-STAT1↓,COX2↓,TNF-α↓	ERK1/2, STAT1, p53	Wistar rat, UB/OC-1 cell	Decrease	Protection	Borse et al. (2017)	2017
Tempol	ROS↓,Cleaved-PARP↓,Cleaved-caspase 3↓, iNOS↓	/	HEI-OC1 cell	Decrease	Protection	Youn et al. (2016)	2016
Dexamethasone (DXM)	Caspase-3、8、9↓,NOX-3↓,ROS↓	NOX-3	Wistar rat, Sprague Dawley rat	Decrease	Protection	Özel et al. (2016), Dinh et al. (2015)	2016 2015
Methylprednisolone	Caspase-3↓,Caspase-8↓,Caspase-9↓	/	Adult female Wistar rat	Decrease	Protection	Özel et al. (2016)	2016
Ceramide-1-phosphate (C1P)	p-Akt↑,p-MAPK↑	PI3-K/Akt,MAPK	C57BL/6J mice	Decrease	Protection	Le et al. (2016)	2016
NVP-231	CERK↓	CERK	C57BL/6J mice	Increase	Injury	Le et al. (2016)	2016
D-α-tocopherol succinate	ROS↓,Caspase-3↓,Cleaved-PARP↓	/	HEI-OC1 cell	Decrease	Protection	Kim et al. (2016)	2016
R-phenylisopropyladenosine (R-PIA)	A1AR↑,ROS↓,NOX3↓,Ser727 p-STAT1↓,iNOS↓,COX-2↓,Cleaved- caspase-3↓.Bcl2↓,p53↓,ERK1/2↓,p38↓,JNK↓	NOX3/STAT1	Wistar rat, UB/OC-1 cell	Decrease	Protection	Kaur et al. (2016)	2016
Hesperetin	MPO↓,MDA↓,TOS↓,OSI↓,TAC↑, ,TUNEL-positive cells↓	/	Wistar rat	Decrease	Protection	Kara et al. (2016)	2016
Ginkgolide B (GB)	NOX2↓,Nrf2↑,HO-1↑,P-Akt↑,Bax↓,Cyto C↓,Cleaved-PARP↓,Caspase 3、9 ↓,ROS↓	NOX2, Akt–Nrf2–HO-1	HEI-OC1 cell, Sprague-Dawley rat	Decrease	Protection	Ma et al. (2015)	2015
Quercetin	ROS↓,Caspase 3、9↓	/	Zebrafish	Decrease	Protection	Lee et al. (2015)	2015
Korean Red Ginseng (KRG)	IL-6↓,Cyto C↓,Caspase 1、3、9↓,Cleaved-PARP↓,NF-κB↓,ROS↓	NF-κB	HEI-OC1 cell, Sprague Dawley rat, Balb/c mice	Decrease	Protection	Kim et al. (2015), Im et al. (2010)	2015 2010
Edaravone (MCI-186, 3-methyl-1-phenyl-pyrazolin-5-one)	ROS↓,Caspase 3↓,Cleaved PARP↓,TUNEL-positive cell↓	/	HEI-OC1 cell, Zebrafish	Decrease	Protection	Im et al. (2015), Hong et al. (2013)	2015 2013
Alpha lipoic acid (α-LA)	ROS↓,Caspase 3↓,p-IκBα↓,IL-1β↓,IL-6↓,p-ERK↓,p38↓	MAPKs Proinflammatory cytokines	Wistar rat, Sprague Dawley rat, HEI-OC1 cell	Decrease	Protection	Altintoprak et al. (2015), Kim et al. (2014)	2015 2014
3-Amino-3-(4-fluoro-phenyl)-1H-quinoline-2,4-dione (KR-22335)	ROS↓,p-JNK↓,p38↓,Cleaved-caspase 3↓,Cleaved-PARP↓	MAPK	HEI-OC1 cell, Zebrafish	Decrease	Protection	Shin et al. (2014)	2014
Dexmedetomidine (DEX)	TUNEL-positive cell↓	/	Zebrafish	Decrease	Protection	Min et al. (2014)	2014
Silymarin	Cleaved caspase 3↓,Cleaved-PARP↓	/	HEI-OC1 cell	Decrease	Protection	Cho et al. (2014)	2014
Metformin	ROS↓,Caspase 3↓,Cleaved-PARP↓,SIRT3 ↑	SIRT3	HEI-OC1 cell	Decrease	Protection	Du et al. (2023), Chang et al. (2014)	2014 2023
Trichostatin A (TSA)	Tnfrsf1a mRNA↓,Ltbr mRNA↓,Tnfrsf11b mRNA↓,Caspase 4、7、12 mRNA↓,Capn1 mRNA↓,Capn2 mRNA↓,CAMK2A mRNA↑,、CAMK2B mRNA↑,Map2k6 mRN↑,Snap25 mRN↑,Vglut1 mRN↑,Rab3b mRN↑	Capn1, Capn2, Tnfrsf1a、Tp53	Wistar rat	Decrease	Protection	Wang et al. (2013)	2013
3-amino-3-(4-fluoro- phenyl)-1H-quinoline-2,4-dione (KR-22332)	ROS↓,TUNEL-positive cell↓,NOX3↓,p53↓,p-ERK↓,p-JNK↓,c-jun↓,Cleaved caspase 3↓,Cleaved PARP↓,TNF-a↓	p53,MAPK	HEI-OC1 cell, Zebrafish, Sprague–Dawley rat (cochlear explant)	Decrease	Protection	Shin et al. (2013)	2013
Gingko biloba extracts (EGb 761)	Caspase-3↓,PARP↓,Cx↑,GJIC↑	GJIC,Cx	HEI-OC1 cell, Sprague–Dawley rat; Wistar rat	Decrease	Protection	Choi et al. (2013a), Huang et al. (2007)	2013 2007
Apocynin	Caspase-3↓,ROS↓,TUNEL-positive cell↓	/	HEI-OC1 cell, Zebrafish	Decrease	Protection	Choi et al. (2013b)	2013
Purple bamboo salt (PBS)	IL-6↓,Caspase-3↓,NF -kB↓,Cyt c↓,ERK↓,Caspase-8↓,Caspase-9↓,Caspase-1↓,ROS↓,AIF↓	NF -kB	HEI-OC1 cell, C57BL/6 mice	Decrease	Protection	Myung et al. (2011), Jeong et al. (2011a)	2011 2011
Etanercept	TNF-α↓	TNF-α	Wistar rat	Decrease	Protection	Kaur et al. (2011)	2011
Rosmarinic Acid	Caspase-1↓,Caspase-3↓,Caspase-9↓,Bax↓,Bcl-2↑,ROS↓,NF -kB↓,Cyt c↓,AIF↓	NF-kB,AIF	HEI-OC1 cell, Spague Dawley rat	Decrease	Protection	Jeong et al. (2011b)	2011
Phloretin	Bcl-2↑,Bax↓,Caspase-3↓、Caspase-8↓、Caspase-9↓,HO-1↑	HO-1	HEI-OC1 cell	Decrease	Protection	Choi et al. (2011)	2011
SB 216763 and LiCl	GSK-3↓,TNF-a↓,IL-1b↓,IL-6 ↓,Caspase-3↓、Caspase-8↓、Caspase-9↓	GSK-3	C57BL/6J and BALB/c mice, HEI-OC1 cell	Decrease	Protection	Park et al. (2009)	2009
Epicatechin	ROS↓,Caspase-3↓	/	HEI-OC1 cell, Zebrafish	Decrease	Protection	Kim et al. (2008)	2008
Piperine	HO-1↑,Nrf2↑	Nrf2–HO-1	HEI-OC1 cell	Decrease	Protection	Choi et al. (2007)	2007

**TABLE 2:** CIO protective agents associated with autophagy.

Compound	Target	Mechanism	Model	Autophagy	Effect	Reference	Year
Trehalose	LC3II↑, P62↑, LAMP1↑, LAMP2A↑, ATG5 mRNA↑, ATG7 mRNA↑, Becn1 mRNA↑, TFEB mRNA↑, Lamp2 mRNA↑, p62 mRNA↑	TFEB	C57BL/6J mice, HEI-OC1 cell	Increase	Protection	Li et al. (2022b)	2022
Metformin	AMPK↑, FOXO3a↑	AMPK/foxo3a	C57BL/6 mice, HEI-OC1 cell, Zebrafish	Increase	Protection	Liang et al. (2021)	2021
U0126	LC3II↓, Beclin 1↓	—	wild-type C57BL/6 mice, HEI-OC1 cell	Decrease	Protection	Wang et al. (2021)	2021
Meclofenamic Acid	LC3II↓	—	HEI-OC1 cell	Decrease	Protection	Li et al. (2018)	2018
Rapamycin	LC3-II/GAPDH↓, Beclin 1↑	—	Wistar rat	Increase	Protection	Fang and Xiao (2014)	2014

**TABLE 3:** CIO protective agents associated with ferroptosis.

Compound	Target	Mechanism	Model	Ferroptosis	Effect	Reference	Year
4-octyl itaconate a	ROS↓,TNF-α mRNA↓,IL-6 mRNA↓,IL-1β mRNA↓,SLC7A11 mRNA↑,GPX4 mRNA↑,PTGS2 mRNA↓,SLC7A11↑,GPX4↑,NRF2↑,HO-1↑,NQO1↑	Nrf2/HO-1	C57BL/6 mice, HEI-OC1 cell	Decrease	Protection	Zhang et al. (2024)	2024
Alpha-lipoic acid (α-LA)	ROS↓, Caspase 3↓, Cleaved-PARP↓, p62↑, LC3-I↑, LC3-II↑, 4-HNE↓, xCT↑, GPX4↑	/	HEI-OC1 cell line	Decrease	Protection	Cho et al. (2022)	2022
Ferrostatin-1 (Ferr-1)	GPX4↑, GSH↑, SLC7A11↑	/	C57BL/6 mice, HEI-OC1 cell	Decrease	Protection	Liu et al. (2024b), Ma et al. (2022)	2021 2020

## 7 Discussion and conclusion

The early detection of cancer and improved treatments have led to an increase in the number of cancer survivors. Therefore, the medical community is becoming increasingly concerned about the quality of life of patients who survive oncological treatments. Cisplatin is widely used to treat various solid tumors. However, ototoxicity induced by cisplatin chemotherapy seriously affects the quality of life of chemotherapy patients, especially children, impacting their speech development and overall physical and mental health. Understanding the molecular basis of cisplatin-induced ototoxicity can help improve hearing loss in chemotherapy patients. Programmed cell death is a critical area of research in biology and medicine. Cisplatin induces ototoxicity through various programmed cell death pathways, including apoptosis, autophagy, ferroptosis, and pyroptosis. Each of these pathways has unique mechanisms and implications for patient management: 1) Apoptosis is a form of programmed cell death characterized by cell shrinkage and DNA fragmentation. It plays a primary role in CIO, and targeting apoptosis may provide therapeutic avenues to protect against hearing loss. 2) Ferroptosis is a recently recognized form of regulated cell death driven by iron-dependent lipid peroxidation. Its involvement in CIO suggests that modulating iron metabolism and oxidative stress may offer protective strategies. 3) Pyroptosis is an inflammatory form of cell death that occurs in response to infection or cellular stress. Understanding its role in CIO could reveal new insights into how inflammation contributes to ototoxicity and how it might be controlled. 4) Autophagy, in contrast, presents a dual role that remains unclear. It can either protect cells by removing damaged organelles and proteins or contribute to cell death under certain conditions. Clarifying whether autophagy acts protectively or destructively in the context of CIO is crucial for developing targeted interventions. This review highlights the molecular mechanisms of programmed cell death in cisplatin-induced ototoxicity (Figures 1–4). We discuss how inhibiting apoptosis, ferroptosis, and pyroptosis can alleviate CIO to some extent, emphasizing the need for a nuanced understanding of these pathways to inform clinical strategies. Moreover, our review synthesizes the latest preclinical findings identifying compounds that provide varying degrees of protection against CIO. Although many of these compounds are yet to be evaluated in clinical trials, the recent FDA approval of sodium thiosulfate for the treatment of cisplatin-induced hearing loss in pediatric patients with nonmetastatic hepatoblastoma marks a significant step forward. However, it is essential to note that sodium thiosulfate may interfere with the anticancer effects of cisplatin and increase its nephrotoxicity, highlighting the need for careful consideration in its application. In summary, this review aims to elucidate the distinct roles of various programmed cell death pathways in cisplatin-induced ototoxicity and their potential implications for clinical practice, thereby providing a comprehensive resource for researchers and clinicians seeking to enhance the quality of life for cancer survivors.

Based on our analysis, we summarize the challenges and research directions for future studies: 1) There are controversies and differences in the RCD pathways involved in cisplatin-induced ototoxicity, and current studies cannot fully reveal the relationship between autophagy and cisplatin-induced ototoxicity. Additional research is needed in this area. 2) Significant advances have been made in understanding cisplatin-induced ototoxicity related to programmed cell death studies. However, clarifying how to integrate the various pathways to understand the detailed ototoxicity mechanisms is a major challenge for future studies. We hypothesize that an upstream pathway might regulate multiple cell death pathways simultaneously. Exploring the link between different modes of cell death and cisplatin-induced ototoxicity is a promising direction for future research. An ideal outcome would be identifying a compound that simultaneously modulates multiple cell death pathways and protects against cisplatin-induced ototoxicity. 3) Can these protective agents be combined with cisplatin without affecting their therapeutic efficacy against tumors? Most studies have been limited to validation in hair cell lines and tumor-free animals. Hearing protection methods need to be tested in animal models with tumors to determine their potential impact on cisplatin-mediated cancer chemotherapy. This could lead to methods to protect normal tissues (e.g., ears and kidneys) without affecting the efficacy of cisplatin chemotherapy and the development of new cisplatin-based cancer therapies. An ideal approach for hearing protection would be to develop a therapy that protects the cochlea while enhancing the therapeutic effects of cisplatin on tumors. 4) The presence of the blood-labyrinth barrier (BLB) in the inner ear, which regulates the ionic composition of the inner and outer lymph, protects the inner ear from blood-borne toxins and selectively allows ions, fluids, and nutrients to enter the cochlea. This may result in the inability of drugs to reach an effective concentration in the inner ear, thus reducing their protective effects after systemic administration (Yu et al., 2020; Gu et al., 2022; Barbara et al., 2022). Identifying an effective mode of drug administration and drug delivery medium is an area that needs to be explored in depth. 5) The complex mechanism of cisplatin-induced ototoxicity cannot be reduced to a single pathophysiological mechanism or the inhibition or activation of a single cell death type. Therefore, a combination of drugs and therapeutic modalities targeting multiple programmed cell death pathways may be a more promising strategy for treating hearing loss. 6) Validating genetic variants associated with cisplatin-induced ototoxicity and understanding their mechanisms may not only determine their impact on cellular signaling pathways but also have significant therapeutic potential.

In conclusion, this review contributes to our understanding of the different functions of PCD in CIO to a certain extent. Studying the PCD pathways involved in CIO has led to breakthroughs in understanding the complex molecular pathogenesis of this severe adverse drug reaction. However, further progress in this area is needed. With a deeper understanding of the critical pathways involved in the development of CIO, new and more effective ways to prevent and treat this condition will emerge.

## Glossary

CIO: cisplatin-induced ototoxicity
OHCs: outer hair cells
SGNs: spiral ganglion neurons
PCD: Programmed cell death
Caspase: cysteinyl aspartate specific proteinase
ER: endoplasmic reticulum
ROS: reactive oxygen species
Cytc: cytochrome C
MMP, ΔΨ: mitochondrial membrane potential
tBid: truncated Bid
XIAP: X-linked inhibitor of apoptosis protein
DR: death receptor
TNFR: tumor necrosis factor receptor
FAS/FASL: Fas cell surface death receptor/Fas ligand
TRAILR/TRAIL: Tumor necrosis factor-related apoptosis-inducing ligand receptor/Tumor necrosis factor-related apoptosis-inducing ligand
TNFR/TNF: Tumor necrosis factor receptor/Tumor necrosis factor
FADD: Fas-associated with death domain protein
UPR: unfolded protein response
PERK: protein kinase RNA-like endoplasmic reticulum kinase
eIF2α: eukaryotic initiation factor 2α
ATF4: activating transcription factor 4
CHOP: C/EBP homologous protein
ATF6: transcription factor 6
TUDCA: taurine deoxycholic acid
Atgs: autophagy-related genes
PARL: presenilin-associated rhomboid-like protease
PINK1: phosphatase and tens of homologues-induced putative kinase 1
BNIP3: Bcl-2/adenovirus E1B 19 kDa interacting protein 3
NLRX1: Nucleotide-binding domain and leucine-rich repeat containing family member X1
DRP1: Dynamin-related protein 1
AMPK: adenosine monophosphate-activated protein kinase
FOXO3: factor forkhead box protein O3
mTORC1: mTOR complex 1
TFEB: transcription factor EB
ACC2: acetyl-coenzyme A carboxylase 2
MA2: meclofenamic acid
NSAID: non-steroidal antiinflammatory drug
Nrf2: Nuclear factor erythroid 2-related factor 2
GSH: glutathione
GPX4: glutathione peroxidase 4
HO-1: heme oxygenase-1
TfR1: transferrin receptor 1
GSDM: Gasdermin
NLRP3: NOD-like receptor protein 3
ASC: apoptosis-associated speck-like protein
TXNIP: Thioredoxin-interacting protein
Trx: TXNIP expression or Thioredoxin
MC: marginal cell
HMG-CoA: hydroxymethylglutaryl-CoA
BLB: blood labyrinth barrier
FDA: the US Food and Drug Administration.
